# Effects of hypoxia inducible factor-1α on apoptotic inhibition and glucocorticoid receptor downregulation by dexamethasone in AtT-20 cells

**DOI:** 10.1186/s12902-015-0017-2

**Published:** 2015-05-23

**Authors:** Chenran Zhang, Qiang Qiang, Ying Jiang, Liuhua Hu, Xuehua Ding, Yicheng Lu, Guohan Hu

**Affiliations:** Department of Neurosurgery, Shanghai Changzheng Hospital, Second Military Medical University, No. 415, Feng-Yang Road, Shanghai, 200003 China; Department of Neurology, Huadong Hospital, Fudan University, Shanghai, 200040 China; Department of Cardiology, Ren Ji Hospital, School of Medicine, Shanghai Jiao Tong University, Shanghai, 200127 China

**Keywords:** Hypoxia inducible factor–1α, Glucocorticoid receptor, Apoptosis, Dexamethasone, ACTH pituitary adenomas

## Abstract

**Background:**

Hypoxia inducible factor-1α (HIF-1α) is the central transcriptional regulator of hypoxic responses during the progression of pituitary adenomas. Although previous immunohistochemical studies revealed that HIF-1α is expressed in adreno-cortico-tropic-hormone (ACTH) pituitary adenomas, the role of HIF-1α remains unclear.

**Methods:**

AtT-20 cells were incubated under hypoxic conditions (1 % O_2_) for 12 h. HIF-1α mRNA and protein expression levels were measured by real-time PCR and western blotting, respectively. BrdU was used to determine the effects of hypoxia on cell viability. AtT-20 cells were transfected with siRNA targeting HIF-1α, followed by hypoxia (1 % O_2_) for 12 h. Apoptosis was determined by annexin V-FITC flow cytometry and Tdt-mediated dUTP nick end-labelling (TUNEL) assay. In addition, we examined interactions between HIF-1α, glucocorticoid receptor (GR), and dexamethasone under both normoxic and hypoxic conditions.

**Results:**

Hypoxia triggered the time-dependent proliferation of AtT-20 cells in association with increased HIF-1α mRNA and protein levels. However, the viability of AtT-20 cells decreased greatly when they were first transfected with HIF-1α-siRNA and then exposed to hypoxia. According to flow cytometry (annexin V-FITC and PI staining) and TUNEL analyses, a greater percentage of cells were apoptotic when transfected with HIF-1α siRNA and subsequently cultured under hypoxic conditions compared to those in the normoxia and mock groups. After AtT-20 cells were cultured in 1 % O_2_ and then treated with dexamethasone, HIF-1α levels significantly increased or decreased in normoxic or hypoxic conditions, respectively. Dexamethasone suppressed GR expression to a higher degree in hypoxic than normoxic conditions. Downregulation of GR by dexamethasone was greatly prevented in cells that were transfected with HIF-1α siRNA.

**Conclusions:**

These findings strongly suggest that HIF-1α exerts an antiapoptotic role and participates in the downregulation of GR by dexamethasone in hypoxic AtT-20 cells.

## Background

Pituitary adenomas are less vascularised [1] and oxygenated [2] compared to the anterior and posterior lobes of the pituitary gland, in contrast to other malignant neoplasms. This phenomenon suggests that pituitary adenoma cells might acquire tolerance to hypoxia. Normally, hypoxia can induce tumour cell death [3]; however, the presence of necrotic or apoptotic changes is rarely observed in pituitary adenomas [4].

Hypoxia inducible factor-1α (HIF-1α) is the central transcriptional regulator of the hypoxic response. Activated HIF-1α drives the transcription of more than 2 % of all human genes either directly or indirectly to adjust the homeostasis of cells under hypoxic conditions [5]. A previous article reported that HIF-1α is expressed in ACTH pituitary adenomas [6]. However, the roles of HIF-1α in the development and clinical presentation of ACTH pituitary adenomas remain unknown.

The relationship between HIF-1α and apoptosis was recently revealed [7]. It remains controversial whether hypoxia induces a pro- or antiapoptotic process [8]. Coincidently, adaptation to hypoxic environments is also influenced by glucocorticoids. There is cross-talk between hypoxia-dependent signals and glucocorticoid-mediated regulation of gene expression [9-11]. However, the cross-talk between HIF-1α and glucocorticoid pathways remains poorly defined.

In the present study, we employed a special hypoxia incubator [12,13] and HIF-1α siRNA to study the effects of HIF-1α expression on the proliferation and protection from apoptosis in AtT-20 cells in vitro. We also explored the interactions of HIF-1α, glucocorticoid receptor (GR), and dexamethasone under both normoxic and hypoxic conditions.

## Methods

### Cell culture and hypoxia induction

AtT-20 cells were obtained from the American Type Culture Collection (Manassas, VA, USA) and routinely cultured in Ham’s-F12K medium (Gibco, USA), supplemented with 2 mM L-glutamine, 1.5 g/L sodium bicarbonate (82.5 %), horse serum, (15 %), and foetal bovine serum (Gibco, BRL) (2.5 %) at 37 °C in a humidified atmosphere containing 5 % carbon dioxide. For hypoxic exposure, cells were cultured in a specifically designed hypoxia incubator (Thermo Electron, Forma, MA) in an atmosphere consisting of 94 % N_2_, 5 % CO_2_, and 1 % O_2_.

### Reagents

Dexamethasone and dimethyl sulfoxide (DMSO) were purchased from Sigma-Aldrich. The short-interfering RNA (siRNA) against mouse HIF-1α and mock siRNA (nonsense sequence) were purchased from Santa Cruz Technology (Santa Cruz, CA). All other reagents were of the highest analytical grade available.

### BrdU

Cell proliferation of AtT-20 cells in hypoxic conditions was measured by a BrdU incorporation assay. Cells under different treatments were plated onto 96-well plates. These cells were subjected to BrdU using the BrdU Cell Proliferation Kit (Cell Signaling, USA) following the manufacturer’s instructions. Three independent experiments were performed. Next, we employed Multisizer 3 COULTER COUNTER to count the AtT20 cell numbers. Before counting the sample, flush the system and count PBS as a blank control. Put 0.2 ml of suspension of cells into 10 ml PBS, and then mix the sample thoroughly. Turn the glass valve directly above the cells to vertical, and then turn it back to horizontal to begin the count. The count displayed is the the number of cells in 0.5 ml.

### RNA extraction and real-time PCR

**Fig. 1:**
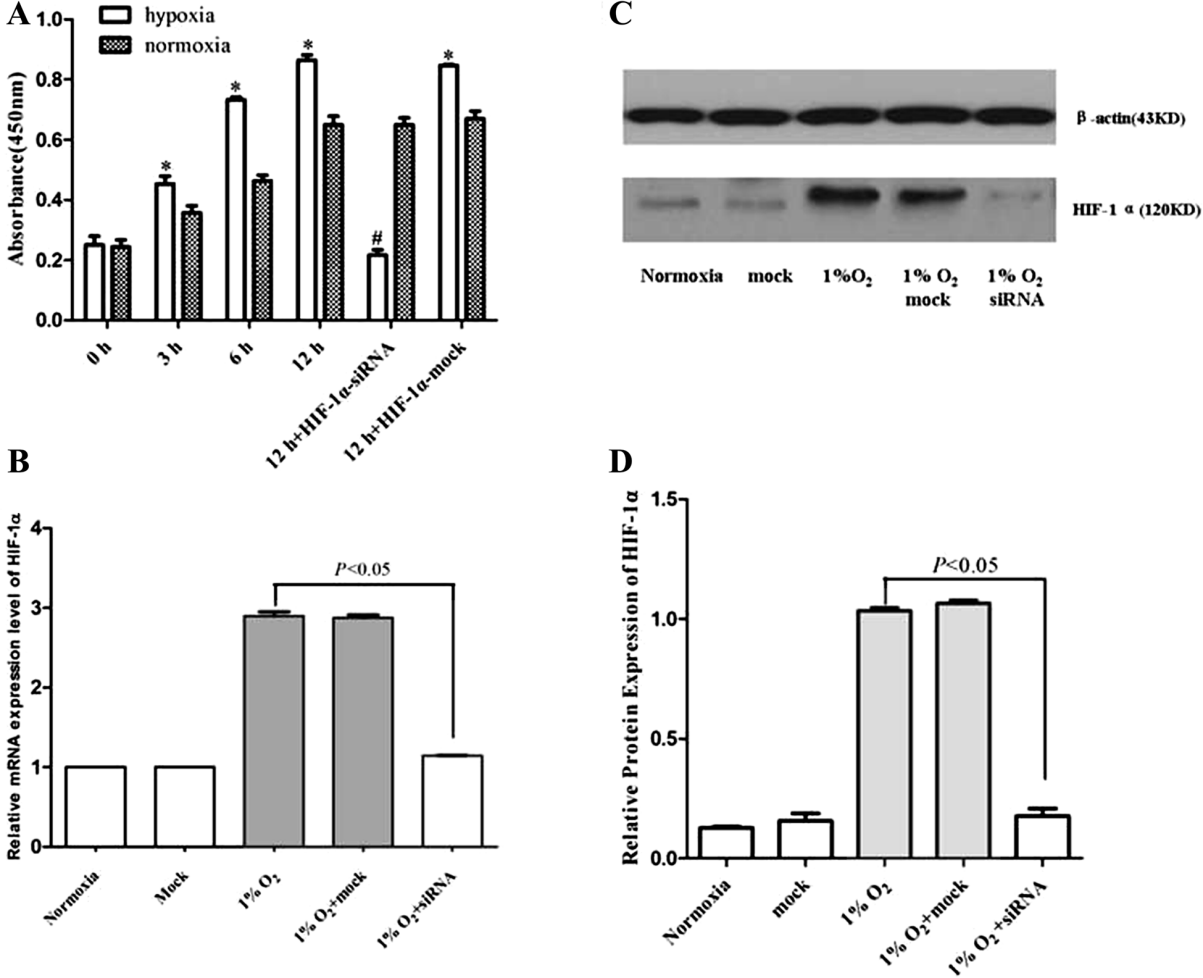
Effects of hypoxia on proliferation of AtT-20 cells. (**a**) AtT-20 cells were incubated under hypoxic (1% O_2_) and normoxia conditions for the indicated times (0, 3, 6, and 12 h). Brdu assay showed hypoxia incubation stimulated the proliferation of AtT-20 cells in a time-dependent manner compared with normoxia group (*P < 0.05 vs. normoxia groups). Cells transfected with HIF-1α-siRNA then cultured in hypoxic incubator for 12 h diminished the BrdU incorporation (#*P* < 0.05 vs. mock). Knock-down of AtT-20 cells with HIF-1α siRNA (10 μM) was evaluated by real-time PCR (**b**) and western blot (**c**) (*P* < 0.05 vs. mock). The hypoxic and normoxia incubation time is 12 h. (**d**) Quantification of (**c**). Independent experiments were repeated at least three times and similar results were obtained

**Fig. 2:**
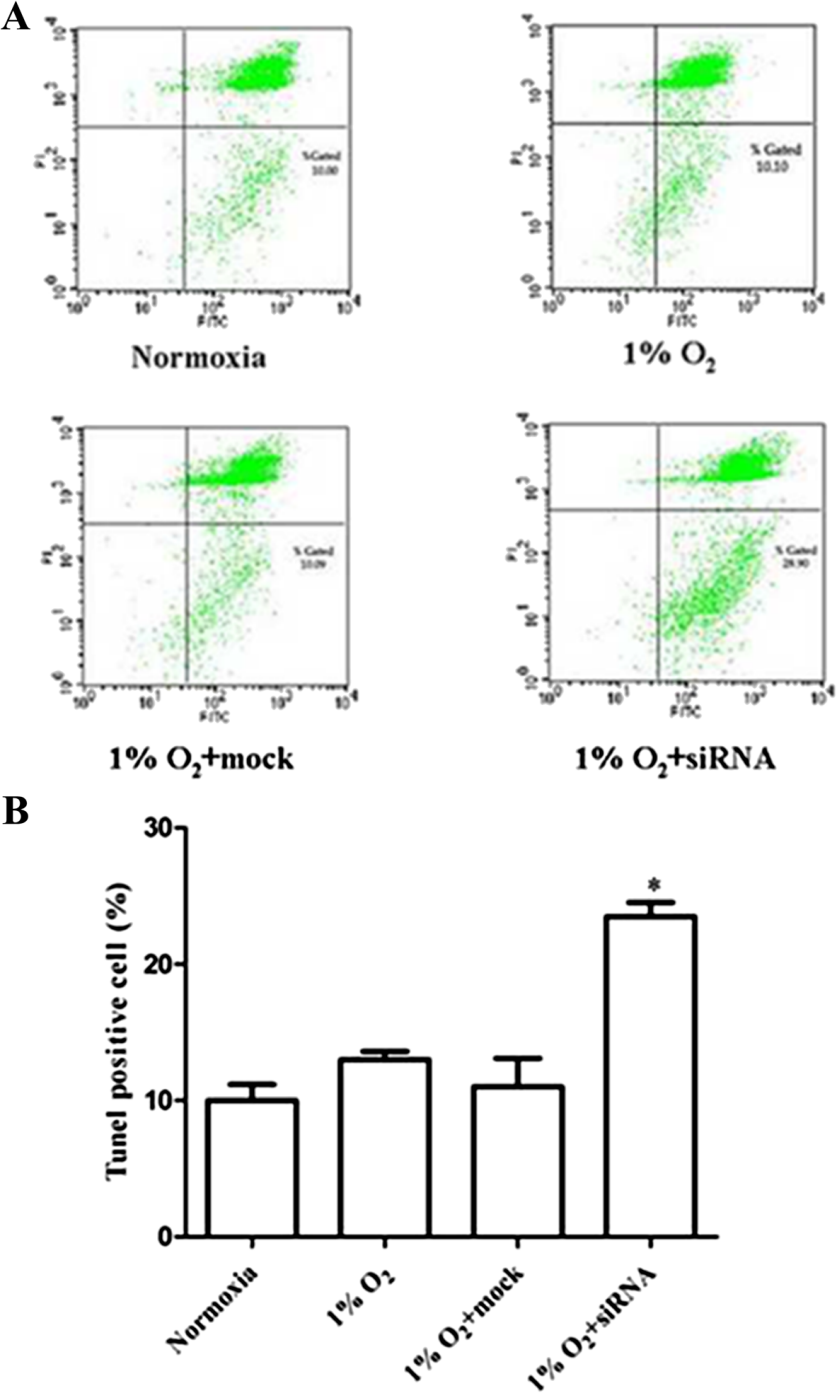
Role of HIF-1α in protecting AtT-20 cells from hypoxia-induced apoptosis. AtT-20 cells were transfected with 10 μM HIF-1α siRNA or mock transfected, and subsequently cultured in hypoxic or normoxic conditions for 12h. (**a**) Annexin V-FITC and (**b**) TUNEL assay showed increased apoptosis when cells were transfected with HIF-1α siRNA and subsequently cultured in 1% O_2_ for 12 h compared with normoxia and mock groups (*P < 0.05 vs.the other groups)

**Fig. 3:**
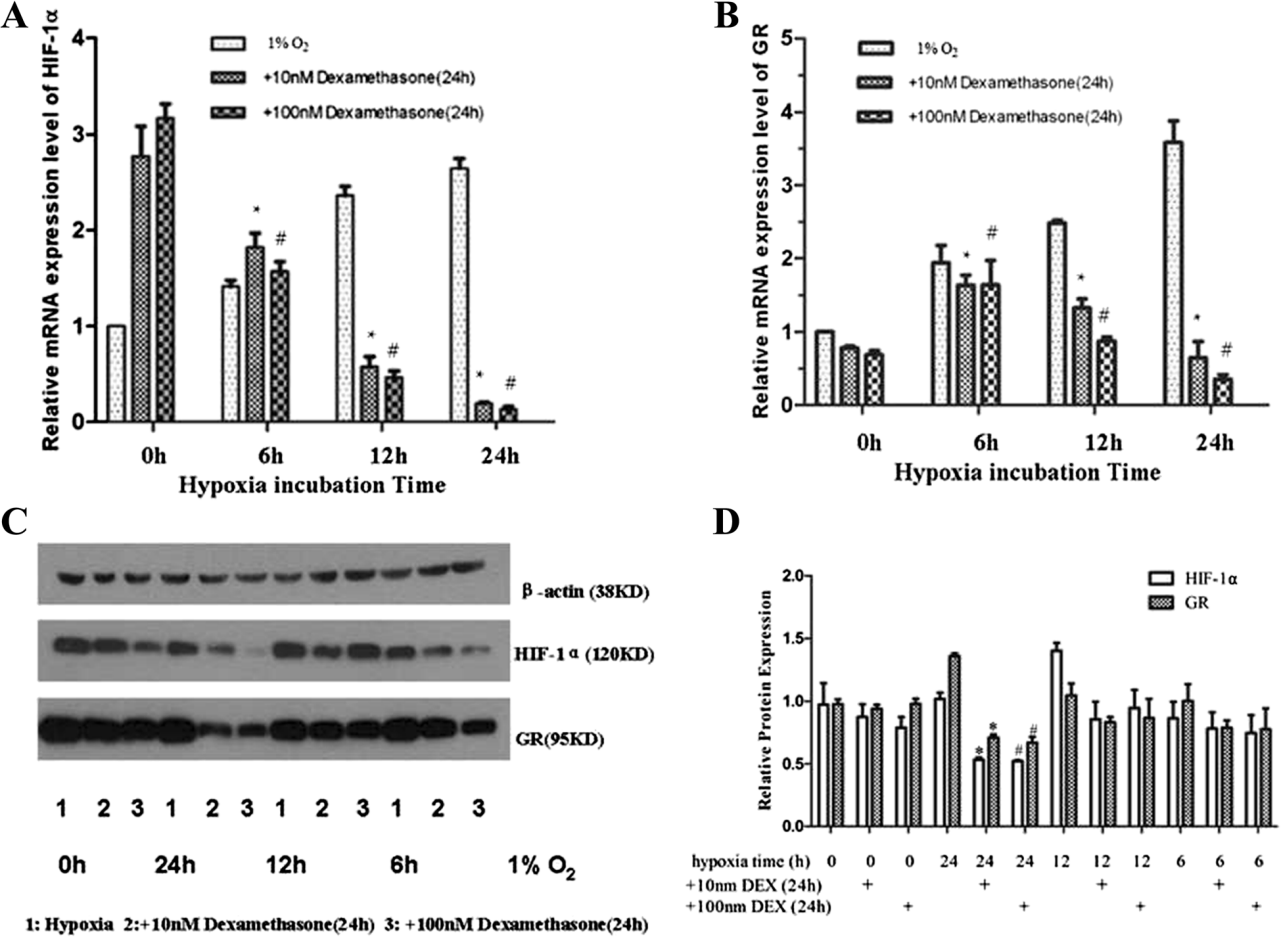
Interaction of HIF-1α, GR, and glucocorticoids after administration of dexamethasone under hypoxic conditions. Effects of dexamethasone (10 nM or 100 nM, 24h) on HIF-1α (**a**, **c**) and GR (**b**, **c**) mRNA and protein expression levels under normoxic and hypoxic conditions. *P < 0.05 vs. 10nM at 0 h. #*P* < 0.05 vs. 100 nM at 0 h. (**d**) Quantification of (**c**) (*,# *P* < 0.05 vs. hypoxia 24 h)

RNA was prepared using the TRIzol reagent (Invitrogen, Carlsbad, CA, USA). One microgram of total RNA, 1 μL oligo(dT) 15 primer (Promega, USA) and DEPC water were added to a total volume of 10 μL, heated to 70 °C for 5 min, and placed on ice for 5 min. Then a mixture of M-MLV RT 5× reaction buffer (5 μL), 100 mM dNTPs (0.5 μL), 1 μL M-MLV RT H(−) point mutant, and DEPC water in a final volume of 15 μL (all from Promega, Madison, USA) was added to each sample, followed by incubation at 40 °C for 60 min and 70 °C for 15 min. Real-time PCR was performed using the SYBR® Premix Ex Taq™ PCR kit (Takara, Japan) on the Applied Biosystems 7300 Real-Time PCR System (Foster, CA, USA). The 20-μL reaction of the SYBR Green assay contained 10 μL of 2× SYBR Premix Ex Taq, 0.4 μL PCR forward primers and 0.4 μL reverse primers, 0.4 μL ROX reference dye (50×), 2 μL cDNA, and 6.8 μL double-distilled H_2_O. PCR was carried out as follows: one cycle of 95 °C for 10 s (pre-denature) and 40 cycles of two steps (95 °C for 5 s and 60 °C for 31 s). At the end of the amplification, a dissociation curve (melting curve) was plotted in the temperature range 65–95 °C. All amplifications and detections were carried out in a MicroAmp optical 96-well reaction plate with optical adhesive covers (Applied Biosystems). PCRs were performed in triplicate, and a reliable internal control under hypoxia, 28S rRNA, was co-amplified to normalize the amount of RNA added to the reaction [14]. Normoxia group in Fig. 1b, 1 % O_2_-0 h without dexamethasone in Fig. 2a and b, 1 % O_2_-0 h in Fig. 3a and b  were used as calibrator for relative quantitative PCR. All data were analysed using the Applied Biosystems 7300 SDS Software (Applied Biosystems, CA, USA). -2^-△△ct^ method was used to analyze the real time PCR results.

Primer sequences were as follows:

- HIF-1α: forward 5’-ACCTTCATCGGAAACTCCAAAG-3’
- reverse 5’-CTGTTAGGCTGGGAAAAGTTAGG-3’;
- GR: forward 5’-AAGAGACAAACGAGAGTCCTTGG-3’
- reverse 5’-GTGTCCGGTAAAATAAGAGGCTT-3’;
- 28S rRNA: forward: 5’- AATGCCTCGTCATCTAAT-3’
- reverse 5’- TTCGCTGGATAGTAGGTA-3’.

We designed the 28S rRNA primers online. The website was sigma.com/probedesignonline. Actually, we had searched many literatures about the 28S primers of mouse, but it was difficult to find a matched one.

### Western blot analysis

Cells were extracted by centrifugation at 3000 rpm for 2 min, followed by one cold PBS wash and lysed in lysis buffer (RIPA 1:1, PMSF 100:1, and protein inhibitor 1:200). After a 30-min incubation on ice, cell lysates were centrifuged at 13000 rpm at 4 °C for 15 min. Total proteins were quantified by the Thermo Scientific Pierce BCA protein assay kit according to its instruction. One hundred micrograms of sample were first electrophoresed on a 7.5 % SDS-polyacrylamide gel and transferred to PVDF membranes. Ponceau S staining was performed on the membranes to ensure successful transfer. After transfer, the membranes were blocked with 10 % fat-free milk for 2 h at room temperature, and then incubated with either rabbit polyclonal anti-HIF-1α antibody (H-206, Santa Cruz) at a 1:500 dilution at 4 °C overnight or anti-GR antibody (M-20, Santa Cruz, CA, USA) at a 1:500 dilution for 2 h at room temperature. After washing three times with 1 × TBS-Tween, the membranes were then incubated for 1 h with anti-rabbit IgG, HRP-linked secondary antibody (HIF-1α, 1:20,000 or GR, 1:3000) and visualized using a chemiluminescence detection kit, ECL-PLUS (Amersham Biosciences). Anti-β-actin (mouse monoclonal, 1:20,000; Calbiochem, La Jolla, CA) was used as protein control. The relative amount of protein was quantified by densitometry using Image J software.

### Knock-down of HIF-1α protein expression with siRNA

AtT-20 cells (4 × 10^5^) were seeded into 12-well plates without antibiotics and incubated at 37 °C for 5 h to 90 % confluence. Four microlitres of 10 μM HIF-1α siRNA (Santa Cruz, CA, USA) and 2 μL Lipofectamine 2000 (Invitrogen, Carlsbad, CA, US) were gently mixed with 100 μL siRNA transfection medium (OPTI-MEM, Gibco, BRL, USA) for 5 min at room temperature, and the mixtures were then combined and incubated at room temperature for another 20 min to form siRNA-Lipofectamine 2000 complexes. The complexes were finally added to the cells. After incubation at 37 °C for 24 h, cells were cultured with medium containing antibiotics and cultured in 1 % O_2_ for 12 h. The efficiency of the HIF-1α knock-down by siRNA was evaluated by real-time PCR and western blot. Mock siRNA (Santa Cruz, CA) was transfected as a negative control.

### Annexin V-FITC detecting system

Apoptotic cells were detected using the annexin V-FITC apoptosis detection kit (BD Biosciences Pharmingen). Cells were seeded into 24-well plates at a density of 40 × 10^4^ cells per well. After a 12-h incubation in hypoxic conditions and transfection with HIF-1α-siRNA, cells were washed twice with cold PBS and then resuspended in 200 μL 1 × binding buffer. One hundred microlitres of the above cell solution were transferred into a 5-mL tube, and 5 μL of annexin V-FITC and 5 μL of PI were added. The tubes were gently vortexed and incubated for 15 min at room temperature in the dark. Four hundred microlitres of 1× binding buffer was added, and the cells were analysed by flow cytometry (BD FACS Calibur) within 1 h. For each measurement 10 000 cells were analyzed.

### Tunel staining

FACS data on apoptosis was further verified using the terminal deoxynucleotidyl transferase (Tdt)-mediated dUTP nick end-labelling (TUNEL) assay. The TUNEL technique was performed using the in situ cell death detection kit (Roche Diagnostics, Indianapolis, IN, USA), according to the manufacturer’s instructions. Chamber slides were fixed with 4 % paraformaldehyde for 1 h, and 0.1 % Triton-100 in 0.1 % sodium citrate was added at 4 °C for 2 min. The slides were incubated with the TUNEL reaction mixture for 1 h at 37 °C. After washing, the slides were further incubated with alkaline phosphatase-conjugated anti-fluorescein antibody for 30 min at 37 °C. Slides were developed using Fast Red (DAKO, Carpenteria, CA, USA) and lightly counterstained with hematoxylin.TUNEL positive cells were observed under confocal microscopy. TUNEL-positive cells per field were counted in 5 random fields under 40× magnifications, and positive cell percentages were averaged.

### Statistical analysis

The two tailed Student’s *t* test was used to analyse the data. Results are expressed as means ± SEM. *P* less than 0.05 was considered statistically significant.

## Results

### Hypoxia triggered the proliferation of AtT-20 cells through the induction of HIF-1α mRNA and protein expression

AtT-20 cells were incubated under hypoxic (1 % O_2_) and normoxia conditions for the indicated time points (0, 3, 6, or 12 h). As shown in Fig. 1a, the BrdU assay showed that hypoxia stimulated the proliferation of AtT-20 cells in a time-dependent manner compared with that of the normoxic group (*P* < 0.05) without any signs of cytotoxicity. Under hypoxic conditions (1 % O_2_) for 12 h, cells transfected with mock siRNA did not show significantly reduced BrdU incorporation compared with untransfected cells. In contrast, cells transfected with HIF-1α-specific siRNA showed significantly diminished BrdU incorporation (Fig. 1a). AtT20 cell numbers (using a Coulter counter) were consistent with the BrdU results (data not shown). These findings were associated with the expression of HIF-1α mRNA and protein, as detected by real-time PCR (Fig. 1b) and western blotting (Fig. 1c and d), respectively.

### Role of HIF-1α in protecting AtT-20 cells from hypoxia-induced apoptosis

To further determine possible mechanisms through which HIF-1α contributes to AtT-20 cell growth and proliferation, RNA interference for HIF-1α was employed by transfecting cells with a specific siRNA against mouse HIF-1α mRNA. After transfecting HIF-1α siRNA, an annexin V-FITC apoptosis detection system was employed to determine whether HIF-1α might shield AtT-20 cells from hypoxia-induced apoptosis. Multiple siRNA concentrations were tested; transfection of 10 μM HIF-1α siRNA for 48 h knocked down hypoxia-induced HIF-1α mRNA and protein expression levels by more than 50 %. The relative mRNA expression levels of HIF-1α according to real-time PCR were 1.00 under normoxic conditions and 2.867 ± 0.3946 in 1 % O_2_ (*P* < 0.05). Relative expression of HIF-1α was significantly reduced to 1.180 ± 0.0694 (*P* < 0.001) by siRNA, whereas mock transfection did not show significant changes in the relative expression of HIF-1α in 1 % oxygen (Fig. 1b). Western blots also showed higher expression of HIF-1α under hypoxic conditions and reduced expression by siRNA (Fig. 1c and d). These experiments indicate a firm gene-silencing effect on HIF -1α by siRNA at the mRNA and protein levels. Annexin V-FITC detection by flow cytometry showed increased early apoptosis when cells were transfected with HIF-1α siRNA and subsequently cultured in 1 % O_2_ for 12 h (28.90 % and 10.00 %, *P* < 0.05) (Fig. 2a) compared with the normoxia and mock groups. These results were confirmed by TUNEL assay. We found that TUNEL-positive cells were weakly expressed in the normoxia and mock groups. However, a significantly greater proportion of cells transfected with siRNA targeting HIF-1α mRNA and cultured in 1 % O_2_ were TUNEL positive (Fig. 2b).

### Interaction of HIF-1α, GR, and glucocorticoids: The role of HIF-1α in GR downregulation after administration of dexamethasone in hypoxic conditions

Next, we tested HIF-1α and GR changes in AtT-20 cells after hypoxic incubation (0, 6, 12, and 24 h) and dexamethasone treatment (24 h). Briefly, AtT-20 cells pretreated with dexamethasone (10 nM or 100 nM) for 1 h were first incubated under hypoxic conditions (1 % O_2_) for the indicated time and then under normoxic conditions for the remaining time. Under normoxic conditions, dexamethasone (10 nM and 100 nM) increased mRNA and protein expression levels of HIF-1α, but had the opposite effect after hypoxic incubation (Fig. 3a, c and d). Dexamethasone suppressed HIF-1α expression in a hypoxia-dependent manner. Downregulation of GR mRNA and protein by dexamethasone was more obvious under hypoxic conditions than under normoxic conditions (Fig. 3b-d).

**Fig. 4:**
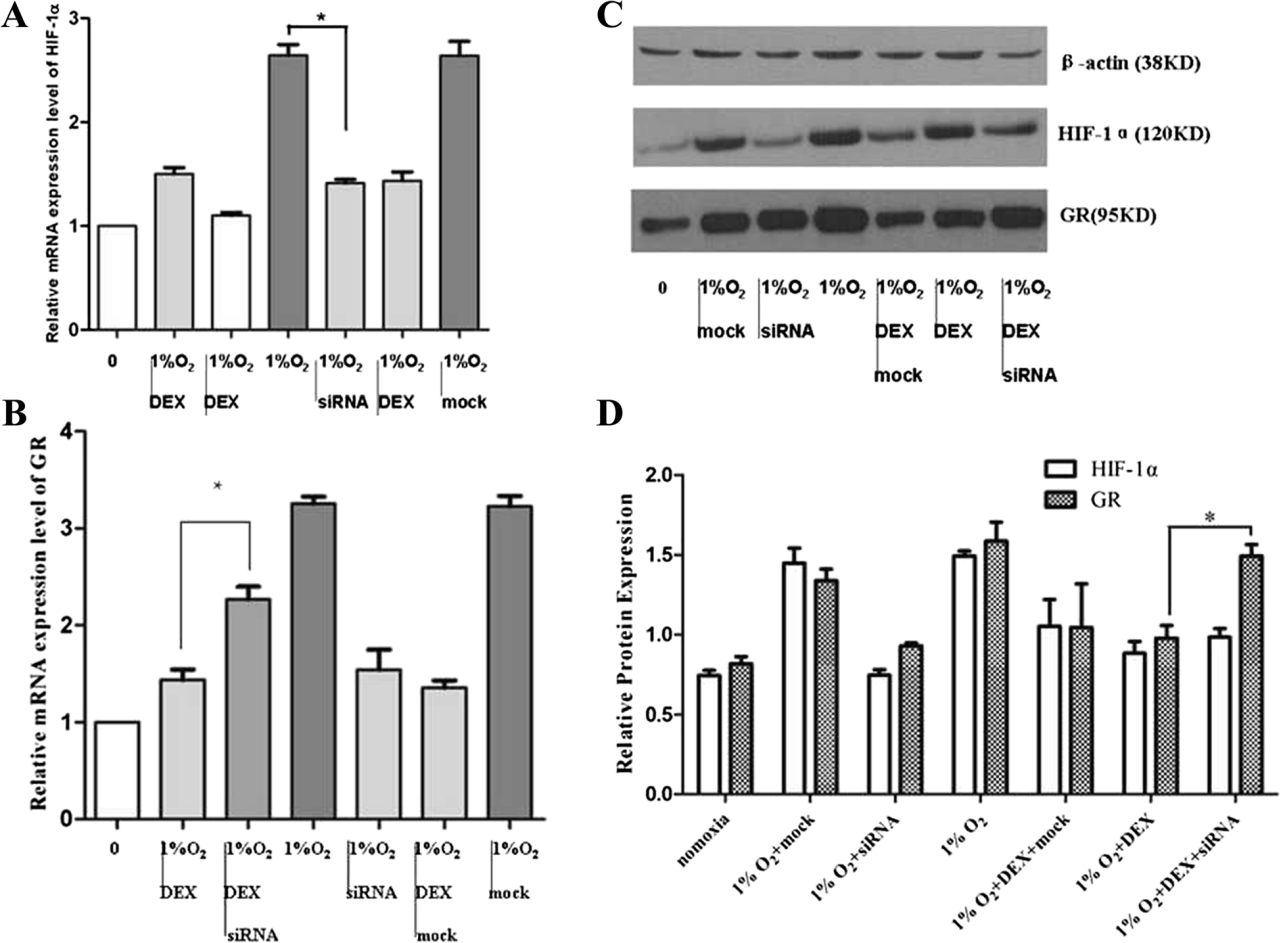
Downregulation of GR by dexamethasone under hypoxia conditions was HIF-1α-dependent. Under normoxia or hypoxic incubation for 12h, mRNA and protein expression levels of HIF-1α (**a**,**c**), GR (**b**,**c**) in cells transfected with HIF-1α-siRNA compared to nontransfected cells after dexamethasone treatment (24h). (**d**) Quantification of (**c**) (*P < 0.05 vs siRNA)

AtT-20 cells were transfected with HIF-1α-siRNA and then cultured in normoxia or hypoxic incubator for 12 h to further clarify whether downregulation of GR by dexamethasone treatment (24h) under hypoxic conditions was HIF-1α dependent. As shown in Fig. 4b-d, mRNA and protein expression levels of GR were increased in cells transfected with HIF-1α siRNA and grown under hypoxic conditions compared to those of nontransfected cells after administration of dexamethasone (*P* < 0.05). In contrast, the mock transfection group exhibited no obvious changes in GR expression levels.

## Discussion

In the current study, we found that hypoxic incubation (1 % O_2_) triggered the growth and proliferation of AtT-20 cells through the induction of HIF-1α expression at the mRNA and protein levels. Transfection of HIF-1α siRNA resulted in the death of most cells. Furthermore, we performed an annexin V-FITC assay to detect early apoptosis and a TUNEL assay to determine the antiapoptotic role of HIF-1α in AtT-20 cells. A greater percentage of cells transfected with HIF-1α siRNA underwent apoptosis compared with that of the mock and hypoxia groups. Thus, we conclude that HIF-1α can protect AtT-20 cells from hypoxia-induced apoptosis. Under normoxic conditions, dexamethasone (10 nM or 100 nM) increased mRNA and protein expression levels of HIF-1α, but suppressed HIF-1α expression under hypoxic conditions. GR expression was upregulated under hypoxic conditions due to increased HIF-1α mRNA and protein levels. Dexamethasone exhibited a stronger suppression of GR expression in hypoxia than in normoxia. Downregulation of GR by dexamethasone was greatly improved in cells transfected with HIF-1α siRNA under hypoxic conditions, implying that dexamethasone-mediated suppression of GR may be HIF-1α dependent.

Whether hypoxia induces a pro- or antiapoptotic response remains unclear [15]. There are published studies in support of both viewpoints. Hypoxia has been shown to indirectly produce a proapoptotic effect by upregulating the expression of Bcl-2 family members, or by associating with and/or stabilizing these proteins [16-19]. However, HIF-1α has also been proposed to have a protective role, limiting hypoxia-induced apoptosis [20-23]. In this regard, pancreatic cancer cell lines that constitutively express HIF-1α are more resistant to apoptosis induced by hypoxia compared to similar cell lines that lack constitutive expression of HIF-1α [24]. Further evidence supporting an antiapoptotic role for HIF-1α is demonstrated by the finding that a neutralising monoclonal antibody against vascular endothelial growth factor (VEGF), the major transcriptional target of HIF-1α, blocks the antiapoptotic effects of hypoxia in HepG2 cells [25]. Yoshida et al. [26] employed microarray analysis to study HIF-1α in the nonfunctional human pituitary adenoma cell line, HP-75, and found that HIF-1α downregulated caspase-10. This finding is consistent with our results, showing that hypoxia-induced HIF-1α increased the growth and proliferation of AtT-20 cells partly due to the role of HIF-1α as an inhibitor of hypoxia-induced apoptosis.

Cross-talk between glucocorticoid and hypoxia-dependent signalling cascades has been demonstrated in several studies. The first evidence of an interaction between HIF-1α and GR was provided by Kodama et al. [9], who found that ligand-dependent activation of GR enhanced hypoxia-dependent gene expression and hypoxia response element (HRE) activity in HeLa cells. Leonard et al. [10] revealed that GR is transcriptionally upregulated by hypoxia in human renal proximal tubular epithelial cells. Further, hypoxic upregulation of GR was confirmed at the level of promoter activity, mRNA, and protein expression, consistent with our results. Wagner et al. [11] demonstrated a dexamethasone-mediated inhibition of HIF-1α target gene expression in hypoxic HEPG2 cells. Furthermore, they showed retention of HIF-1α in the cytoplasm, suggesting a block in nuclear import. Gaber et al. [27] found a clear inhibition of HIF-1α protein expression, which resulted in reduced HIF-1 target gene expression, including VEGF. Interestingly, they also found that PHD1 was regulated in a similar manner as VEGF. Del Peso et al. [28] showed that PHD1 was upregulated by hypoxia in HeLa cells. Therefore, we conclude that the interaction between the glucocorticoid- and hypoxia-dependent signalling cascades is significant as well as varied. These data reflect the complexity and context-specific alterations associated with the differential activation of these cascades in complex tissue microenvironments.

Our study demonstrated that GR expression levels were upregulated by HIF-1α under hypoxic conditions. However, treatment with dexamethasone caused these levels to decrease in a hypoxia-dependent manner. The results clearly indicate that HIF-1α may play a role in the downregulation of GR expression by dexamethasone, which was further confirmed by transfection of HIF-1α siRNA. However, additional experimental evidence is needed to determine the underlying mechanism involved. We are now attempting to employ a luciferase assay, EMSA, and coimmunoprecipitation to clarify the mechanism, and an ACTH pituitary adenoma animal model is under construction.

The current study has profound clinical importance and supports a therapeutic role for HIF-1α in ACTH pituitary adenoma due to its antiapoptotic effects and downregulation of GR. Therefore, we suggest that siRNA targeting HIF-1α may have potential therapeutic value in treating ACTH pituitary adenoma.

## Conclusions

Our present data demonstrate an increased cell proliferation index in AtT-20 cell lines after hypoxia exposure through the induction of HIF-1α expression, which played a crucial role in protecting AtT-20 cells from hypoxia-induced apoptosis. HIF-1α upregulated GR expression at both mRNA and protein levels. We also found that the downregulation of GR by dexamethasone in hypoxic conditions is probably due to the suppression of HIF-1α by dexamethasone, which means that GR downregulation by dexamethasone is HIF-1α dependent.

## Abbreviations

HIF-1α: Hypoxia inducible factor-1
ACTH: Adrenocorticotrop(h)ic hormone
VEGF: Vascular endothelial growth factor
FITC: Fluorescein isothiocyanate
TUNEL: TdT-mediated dUTP-biotin nick end labeling
SDS-PAGE: Sodium dodecyl sulfate polyacrylamide gel electrophoresis
